# Hints to the People upon the Profession of Medicine

**Published:** 1852-01

**Authors:** 


### ARTICLE IX.

*Hints to the People upon the Profession of Medicine.* By WM. MAXWELL WOOD, M. D., Surgeon U. S. Navy, &c. Buffalo: George H. Derby and Co. (From the Publishers through Hewson & Denison, Chicago.)

This is a well written, sound and clear argument in favor of a better appreciation of medical science and more respect for medical men on the part of the public.

As it is furnished in quantities at a low price ($1,50 per dozen) it might not be a waste of means for physicians and societies to purchase and circulate it in their several communities.

